# Want to Know, You Know

**Published:** 1890-08

**Authors:** Frank Kraft

**Affiliations:** Sylvania, Ohio


### "WANT TO KNOW, YOU KNOW."

FRANK KRAFT, M. D., SYLVANIA, OHIO.

A young lady, æt. twenty-two, unmarried, thin and spare, chlorotic and anæmic, intellectual predominance, " mild, gentle, and yielding," has an uncontrollable appetite for raw potatoes. These she must eat before breakfast. They do not distress her. If she overloads, of course, the usual discomfort. Generally constipated. Eyes weak. Left kidney seat of soreness. Some uric acid deposit. Unable to go up-stairs without palpitation. Disposition to faint. Coldness of feet. Skin dry and coolish. Hair, chestnut; same for eyes. Has become convinced that she has tape-worm, and comes to me for such treatment.

The only remedy that I find (in W. Jefferson Guernsey) that has desire for raw potatoes, is Calc-carb.; but with the exception of that one symptom, and the dubious one of inability to go up-stairs, there is not, so far as I can judge, another prominent symptom of Calc-carb. in the case. Alumina looms up as a potato remedy—or rather a remedy the provers of which were made worse by eating potatoes; the anæmia, the chlorosis, the constipation, the *unnatural* appetite, almost persuaded me to give Alumina. Then I turned to Lycopodium, found constipation, satiety from a few mouthfuls, tympany; brick-dust sediment, pain in kidney relieved by urinating, likes oysters, but they distress her unduly, worse of her general symptoms in latter part of afternoon. Her mother, who was present, filled in one of the gaps of silence by saying that she (the mother) had so much wind colic. Every afternoon, clothes got so tight, and that Mary was a good deal like herself. The mother's symptoms were unmistakably Lycopodium. I reviewed my notes carefully, and mentally wished for Dr. Hale's philosophical temperament, so I might cut this Gordian knot by giving, alternately Alumina and Lycopodium, with a possible intercurrent of Calcarea. I gave, instead, Lycopodium, and requested to see patient again in ten days. Was I right?

Why didn't I give Filix-mas, or some other tape-worm specific, and expel the "critter"? Well, first, because I am not sure there is a tape-worm; second, because I am prescribing for my patient, and not for a possible tape-worm; and, third, if Lycopodium is the simillimum it will not only expel the worm, but destroy the eggs and the nutriment for other incipient worms. But suppose Lycopodium is not the simillimum? Then, truly, I have blundered, and must try again; but I am confident my patient will return to me in ten days improved in many ways, even if the raw-potato diet still continues a necessity. Then I shall restudy my case, and again give the nearest simillimum. And still do nothing to expel that worm? Why, yes, the simillimum is at work on that worm. But, ultimately, you will have to take that worm by his horns, so to speak, and expel him, say you. Perhaps. But if I do, it will be because of my ignorance, and I shall sing very small about the performance.

I have seen "worm" tablets given to children, and in due course a large teacupful of wriggling worms was expelled. That's the business, say you. Yes, and I have heard the anxious parent and the modest(?) neighborhood exclaim, "None of your 'sweet sand' medicine for my children. I want to see the worms." And they usually do, for in the course of a fortnight the child is again "wormy," and more tablets are given, and more worms come away, and more satisfaction on the part of the mother. But, by and by, this becomes monotonous; the worms seem to thrive, and for one that comes away a dozen new ones spring into existence. Then, as the child is not improving, but looks wretched and puny, the mother bethinks her of her "little-pill" neighbor, whose child seemed also to be wormy, but, after a course of homœopathic powders, ceased to give any signs of worms, and has since grown strong and fleshy. She visits her friend, and is prevailed on to try some of the "faith" medicine. The result is satisfactory.

In brief: if there is a tape-worm in this case, I propose to make his surroundings so disagreeable to him and his progeny that he and they will be glad to vacate the premises; and if, under pressure, I give way to any peremptory method of expelling his High Nobility, I shall feel that I have descended from my exalted position as a homœopathic physician to the level of that loud advertising glib-mouthed fakir—The Tape-worm King. Am I right?

